# Advances on (+)-nootkatone microbial biosynthesis and its related enzymes

**DOI:** 10.1093/jimb/kuab046

**Published:** 2021-07-19

**Authors:** Xiao Li, Jing-Nan Ren, Gang Fan, Lu-Lu Zhang, Si-Yi Pan

**Affiliations:** Key Laboratory of Environment Correlative Dietology, Ministry of Education, College of Food Science and Technology, Huazhong Agricultural University, Wuhan 430070, China; Key Laboratory of Environment Correlative Dietology, Ministry of Education, College of Food Science and Technology, Huazhong Agricultural University, Wuhan 430070, China; Key Laboratory of Environment Correlative Dietology, Ministry of Education, College of Food Science and Technology, Huazhong Agricultural University, Wuhan 430070, China; College of Food Science and Technology, Henan University of Technology, Zhengzhou 450001, PR China; Key Laboratory of Environment Correlative Dietology, Ministry of Education, College of Food Science and Technology, Huazhong Agricultural University, Wuhan 430070, China

**Keywords:** (+)-Nootkatone, Natural flavor, (+)-Valencene, Nootkatol, Biosynthesis

## Abstract

(+)-Nootkatone is an important functional sesquiterpene and is comprehensively used in pharmaceutical, cosmetic, agricultural and food flavor industries. However, (+)-nootkatone is accumulated trace amounts in plants, and the demand for industry is mainly met by chemical methods which is harmful to the environment. The oxygen-containing sesquiterpenes prepared using microbial methods can be considered as “natural.” Microbial transformation has the advantages of mild reaction conditions, high efficiency, environmental protection, and strong stereoselectivity, and has become an important method for the production of natural spices. The microbial biosynthesis of (+)-nootkatone from the main precursor (+)-valencene is summarized in this paper. Whole-cell systems of fungi, bacteria, microalgae, and plant cells have been employed. It was described that the enzymes involved in the microbial biosynthesis of (+)-nootkatone, including cytochrome p450 enzymes, laccase, lipoxygenase, and so on. More recently, the related enzymes were expressed in microbial hosts to heterologous produce (+)-nootkatone, such as *Escherichia coli, Pichia pastoris, Yarrowia lipolytica*, and *Saccharomyces cerevisiae*. Finally, the development direction of research for realizing industrialization of microbial transformation was summarized and it provided many options for future improved bioprocesses.

## Introduction

Sesquiterpenoids constitute a structurally different category in terpene compounds and possess multiple biological activities, which are oxygen-containing derivatives of sesquiterpenes (Gershenzon & Dudareva, 2007). (+)-Nootkatone is a sesquiterpene compound, which belongs to bicyclic sesquiterpene ketone of *Yashilane series*. It was originally separated from the Nootka cypress tree and trace amount was found in grapefruit later (Macleod, 1965). Afterwards, (+)-nootkatone was found in peel oils from orange, grapefruit, lemon, mandarin, and so on (Gliszczynska et al., 2011). (+)-Nootkatone has a pleasant grapefruit flavor in orange and grapefruit juice and is used commercially as a spice or flavor ingredient (MacLeod Jr & Buigues, 1964; Sauer et al., 2003). It was reported that (+)-nootkatone tasted slightly bitter and had a low odor threshold of about 0.8 ppm in water and 30 ppm in air (Haring et al., 1972; Shaw & Wilson, 1981). (−)-Nootkatone has the higher odor threshold and less overall bioactivity, so it has little commercial interest. While (+)-nootkatone was more extensively used in the food and cosmetic industries, which providing extremely popular flavor and fragrance compounds (Wriessnegger et al., 2014).

(+)-Nootkatone appears to be safe to humans and other mammals and it is regarded as a GRAS substance by FDA (Laine, 2019). It is comprehensively used in the fragrance, food, cosmetics, and medicine applications because of its specific odor activity (Gliszczynska et al., 2011). In recent years, the researches on relevant physiological effects of (+)-nootkatone have become increasingly critical. (+)-Nootkatone plays an important role in fruit defense and it is an attractant for the animals that feed on the ripe fruit (Sharon‐Asa et al., 2003). It has also been clarified that a grapefruit essential oil containing (+)-nootkatone decreases the proportion of somatic fat (Furusawa et al., 2005a). The studies have found that (+)-nootkatone has extensive benefits, for example anti-inflammatory responses (Bezerra Rodrigues Dantas et al., 2020; Chang & Lee, 2016; Choi et al., 2014; Kurdi et al., 2018), AMPK activation (Hung et al., 2019), antibacterial (Farha et al., 2020; Yamaguchi, 2019), and anti-insect properties (Guo et al., 2019; Pérez Del Pulgar et al., 2019; Zhu et al., 2001). (+)-Nootkatone is also an antitumor compound with antiproliferative, proapoptotic activity and beneficial protective effect (Nemmar et al., 2018; Yoo et al., 2020), which inhibits the anticancer growth of retinoblastoma cells (Zhu et al., 2020). Furthermore, (+)-nootkatone possess potential therapeutic treatment effect for neuroinflammation and Alzheimer's disease (Qi et al., 2020; Wang et al., 2018), which also possess neuroprotective effects (He et al., 2018; Qi et al., 2019).

The main production methods for terpenoids include physical extraction (Jaiswal et al., 2014), synthesis of chemical methods (Zhang et al., 2016) and biological catalytic transformation. Generally, the content of terpenoids is very low in plants and the growth of plants is easily affected by climate and environment. The method of plant extraction is easy to destroy wild plant resources seriously. At present, it mainly relies on chemical synthesis of (+)-nootkatone to meet industrial demand. Some unsafe oxidizing agents such as *tert*-butyl peracetate (Wilson & Shaw, 1978) or *tert*-butyl hydroperoxide was used to catalyze (+)-valencene to synthesize (+)-nootkatone (Salvador & Clark, 2002). Hong et al. found that hydrogen peroxide and amphiphilic molybdate ions could be used to catalyze (+)-valencene to synthetize (+)-nootkatone (Hong et al., 2016). It was also found that (+)-valencene could be oxidated by metalloporphyrins to produce (+)-nootkatone for the first time (de Melo et al., 2021). In contrast, the biocatalytic transformation method is not limited by the raw materials (Zhang et al., 2020). The production process is environmentally friendly, sustainable and pollution free, which has obvious advantages (Gounaris, 2010). Biotransformation is a useful substitute for chemical synthesis because of its remarkable regioselectivity and enantioselectivity (Sales et al., 2018; Tai et al., 2016). It is widely used in organic synthesis, spice production, new drug development, modification, and modification of drug structure and prediction of drug metabolism model. Effective European law defined that “natural flavoring substance” as a compound “obtained by appropriate physical methods, enzymes or microbial processes from animals, plants, or microorganism” (Leonhardt & Berger, 2015). Biotransformation processes meets the Europe and the U.S. legal requirements for the “natural” properties of a flavor compound (Kolwek et al., 2018). Therefore, the use of microbial transformation to produce spices has become a hot topic.

The microbial transformation of (+)-valencene has become an increasingly important way to obtain (+)-nootkatone. Therefore, this paper focuses on the recent studies on the microbial synthesis of (+)-nootkatone. We introduce the microbial species and related enzymes involved in the microbial transformation of (+)-valencene to (+)-nootkatone and the heterologous expression to synthesize (+)-nootkatone. Finally, the development direction of the industrialization of microbial transformation is summarized in order to provide reference for the future research of sesquiterpenes biotransformation.

## Precursor Compounds for (+)-Nootkatone Synthesis

### (+)-Valencene as the Precursor

**Fig. 1.**
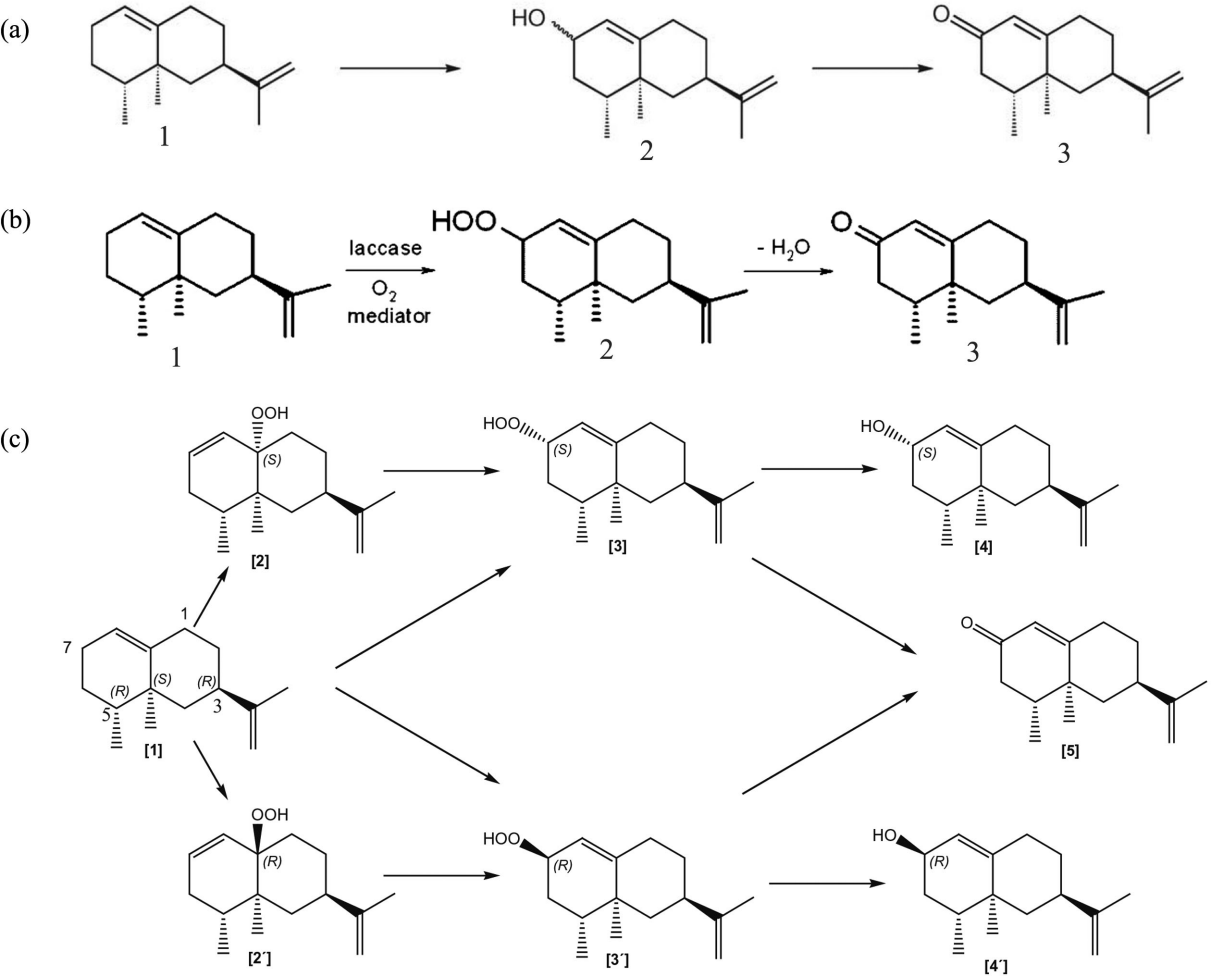
(a) Conversion of (+)-valencene to (+)-nootkatone via nootkatol. 1: (+)-Valencene; 2: nootkatol (β-nootkatol and α-nootkatol); 3: (+)-nootkatone. (b) Transformation of (+)-valencene to (+)-nootkatone by laccase via valencene-2-hydroperoxide (Fraatz et al., 2009). It was followed by a subsequent degradation step by heating or addition of chemical catalysts. 1: (+)-Valencene; 2: valencene-2-hydroperoxide; 3: (+)-nootkatone. (c) Transformation of (+)-valencene to (+)-nootkatone by (+)-valencene dioxygenase from *P. sapidus* via (+)-valencene hydroperoxides [2,2′,3,3′] (Krugener et al., 2010). [1] (+)-Valencene; [2] 2(*R*)-isopropenyl-8(*R*),8a(*S*)-dimethyl-1,3,4,7,8,8a-hexahydro-2*H*-naphthalen-4a(*R*)-yl-hydroperoxide; [2′] 2(*R*)-isopropenyl-8(*R*),8a(*S*)-dimethyl-1,3,4,7,8,8a-hexahydro-2*H*-naphthalen-4a(*S*)-yl-hydroperoxide; [3] 6(*R*)-isopropenyl-4(*R*),4a(*S*)-dimethyl-2,3,4,4a,5,6,7,8-octahydro-naphthalen-2(*S*)-yl-hydroperoxide; [3′] 6(*R*)-isopropenyl-4(*R*),4a(*S*)-dimethyl-2,3,4,4a,5,6,7,8-octahydro-naphthalen-2(*R*)-yl-hydroperoxide; [4] β-Nootkatol; [4′] α-nootkatol; [5] nootkatone.

(+)-Valencene is a natural bicyclic sesquiterpene, which possesses a variety of biological activities. It was found in various citrus species, such as the sweet orange (*Citrus sinensis*) (Frohwitter et al., 2014; Hunter & Brogden Jr, 1965). Usually, (+)-valencene was isolated from citrus oils by steam distillation (Beekwilder et al., 2014). Furthermore, previous study found that (+)-valencene was biosynthesized by introducing (+)-valencene synthases in *Saccharomyces cerevisiae* (Paulino et al., 2020). (+)-Valencene was commercially used as an additive in drinks and flavor food because of its fruity and woody flavor. The market sales volume of (+)-valencene was about 10,000 kg every year (Beekwilder et al., 2014). The enzymatic steps from (+)-valencene to (+)-nootkatone was not entirely clear in current research. It was suggested that the (+)-valencene was transformed to nootkatol through a regioselective allylic hydroxylation and nootkatol was then oxidized to (+)-nootkatone (Fig. 1a) (Drawert et al., 1984). Both of the two steps could be catalyzed by a single multifunctional hydroxylase or oxidase and it may also be a sequential enzyme-mediated reaction (Fraatz et al., 2009).

### Cyclohexane Derivatives as the Precursor

Previous study found that the chemical synthesis of (+)-nootkatone began with 4-acetyl-1-ethoxycylohexen and dimethyl γ-ketopimelate (Marshall & Ruden, 1971; Pesaro et al., 1968). Later, it was discovered that the synthesis of (+)-nootkatone could also be achieved by other cyclohexane derivatives (Dastur, 1973). Yanami et al. reported a method of the synthesis of (+)-nootkatone from the readily available (−)-β-pinene via six steps (Yanami et al., 1979). A facile stereoselective synthesis of (+)-nootkatone has been achieved starting with (+)-nopinone (Yanami et al., 1980). It was found that (+)-nootkatone was obtained from (+)-nopinone with a yield of 14%. Afterwards, 4β,4αβ-dimethyl-Δ6,7-octalin-1-one ethylene acetal was reported to synthesize (±)-nootkatone and (±)-valencene (Torii et al., 1982). Majetich et al. reported a method of synthesize (±)-nootkatone and (±)-valencene from 5-methyl-3-ethoxy-2-cyclohexenone (Majetich et al., 1985).

## Microbial Transformation of (+)-Valencene to (+)-Nootkatone

### Biotransformation of (+)-Valencene by Fungi

Fungi are commonly used in biotransformation of (+)-valencene (Table 1). They are a kind of effective biotransformation carrier because of their variety, low nutritional requirement, and easy to culture. It was found that lignin peroxidase of *Phanerochaete chrysosporium* were able to transform (+)-valencene to (+)-nootkatone in concentrated culture supernatants (Willershausen & Graf, 1991). (+)-Valencene was directly added for conversion as a substrate. The fungal strain *Mucor* sp. was inoculated and cultivated on the czapek-pepton medium and then (+)-valencene was added to the medium. The result showed that 82% (328 mg/l) yield of (+)-nootkatone was obtained (Asakawa et al., 2013; Furusawa et al., 2005b). Another study found that two plant pathogenic fungi *Botryosphaeria dothidea* and *Botryodiplodia theobromae* separated from fruits could also transform (+)-valencene to (+)-nootkatone. (+)-Nootkatone was obtained with the yield of 42–84% (168/l–336 mg/l) subsequently (Asakawa et al., 2013; Furusawa et al., 2005b). The ascomycete *Chaetomium globosum* in submerged cultures was found to transform (+)-valencene to (+)-nootkatone through α-nootkatol in 3 days with a yield of 25 mg/l (+)-nootkatone (Kaspera et al., 2005, Meng et al., 2020). Lyophilisates and homogenized fresh mycelium of *Pleurotus sapidus* was found to produce (+)-nootkatone from (+)-valencene (Fraatz, 2007; Fraatz et al., 2008, 2009; Rüdiger Kaspera, 2004). *Kluyveromyces marxianus NCYC1429, Rhyzomucor* sp., and *Aspergillus tamarii V12307* were cultivated on solid medium with only a low bioconversion percentage of (+)-valencene (Palmerín-Carreño et al., 2015). Three fungal strains *B. theobromae 1368, P. Chrysosporium*, and *Yarrowia lipolytica 2.2ab* were reported that could oxidize (+)-valencene to (+)-nootkatone effectively. *B. theobromae 1368* and *P. chrysosporium* transformed (+)-valencene to (+)-nootkatone with a yield of 231.7 ± 2.1 and 100.8 ± 2.6 mg/l, respectively. It was investigated that the bioconversion of (+)-valencene finally produced 852.3 mg/l (+)-nootkatone by *Y. lipolytica 2.2ab* in a partitioning bioreactor. The use of orange essential oil to divide the three-phase system overcomes the product inhibition (Palmerin-Carreno, Rutiaga-Quinones, et al., 2016). Moreover, they designed the three-phase partitioning bioreactor and four-phase partitioning bioreactor to produce (+)-nootkatone from (+)-valencene by *Y. lipolytica 2.2ab* (Castillo-Araiza et al., 2017; Palmerín-Carreño et al., 2016). In addition, Li et al. optimized the catalytic conditions of the biotransformation of (+)-valencene into (+)-nootkatone by *Y. lipolytica* in shake flasks and the maximum production of (+)-nootkatone reached 628.41 ± 18.60 mg/l (Li et al., 2021).

**Table 1.** Microbial Biotransformation of (+)-Valencene to (+)-Nootkatone

Strain	Time	Nootkatone (maximum yield)	References
**Fungi**			
*Phanerochaete chrysosporium*	/	/	Willershausen & Graf (1991)
*Laccases* from *Botrytis cinerea*	2 days	1296 mg/l	Huang et al. (2001)
*Mucor species*	7 days	82% (328 mg/l)	Asakawa et al. (2013), Furusawa et al. (2005b)
*Botryosphaeria dothidea*	7 days	84% (336 mg/l)	Asakawa et al. (2013), Furusawa et al. (2005b)
*Botryodiplodia theobromae*	7 days	42% (168 mg/l)	Asakawa et al. (2013), Furusawa et al. (2005b)
*Chaetomium globosum*	3 days	25 mg/l	Kaspera et al. (2005)
			Meng et al. (2020)
Lyophilisates of *Pleurotus sapidus*	13 hr	250 mg/l	Kaspera (2004)
*Pleurotus sapidus*	24 hr	320 mg/l	Fraatz (2007), Fraatz et al. (2009)
Homogenised fresh mycelium of *Pleurotus sapidus*	42 hr	600 mg/l	Fraatz et al. (2008), Zorn et al.(2009)
*Botryodiplodia theobromae 1368*	12 days	239.7 ± 2.1 mg/l	Palmerín-Carreño et al. (2015)
*Phanerochaete chrysosporium*	12 days	110.3 ± 11.8 mg/l	Palmerín-Carreño et al. (2015)
*Kluyveromyces marxianus NCYC1429*	12 days	14.51 ± 0.83 mg/l	Palmerín-Carreño et al. (2015)
*Aspergillus tamarii V12307*	12 days	4.70 ± 0.56 mg/l	Palmerín-Carreño et al. (2015)
*Rhyzomucor species*	12 days	0.315 ± 0.23 mg/l	Palmerín-Carreño et al. (2015)
*Yarrowia lipolytica 2.2ab*	4 days	852.3 mg/l	Palmerin-Carreno, Castillo-Araiza, et al. (2016), Palmerin-Carreno, Rutiaga-Quinones, et al. (2016)
**Bacteria**			
*Enterobacter species*	**/**	12%	Dhavlikar and Albroscheit (1973)
*Rhodococcus species*	5 days	0.5 mol% (50 mg/l)	Huang et al. (2001), Okuda, Sonohara, Takigawa, et al. (1994)
*Pseudomonas putida*	**/**	47%	Sowden et al. (2005)
*Bacillus megaterium*	**/**	7.7%	Sowden et al. (2005)
**Microalgae**			
*Chlorella fusca* var. *vacuolata IAMC-28*	18 days	63% (252 mg/l)	Furusawa et al. (2005b)
*Chlorella fusca*	14 days	63% (252 mg/l)	Asakawa et al. (2013)
*Chlorella pyrenoidosa*	14 days	80% (320 mg/l)	Asakawa et al. (2013)
*Chlorella vulgaris*	14 days	90% (360 mg/l)	Asakawa et al. (2013)
**Plant cells**			
*Citrus tissue cultures*	/	trace	Drawert and Berger (1983)
*Citrus paradisi*	6 hr	1.1 mg/l	Drawert et al. (1984)
*Citrus paradisi*	/	2%	Delrio et al. (1991)
*Citrus limonia*	/	2%	Delrio et al. (1991)
*Citrus aurantium*	/	2%	Delrio et al. (1991)
*Gynostemma pentaphyllum*	20 days	72% (650 mg/l)	Sakamaki et al. (2005)
			Leonhardt and Berger (2015)
*Caragana chamlagu*	20 days	25% (225 mg/l)	Sakamaki et al. (2005)
*Hibiscus cannabinus*	20 days	28% (252 mg/l)	Sakamaki et al. (2005)

“/” represent there is no reports in literature.

### Biotransformation of (+)-Valencene by Bacteria

It was reported that the biotransformation of (+)-valencene to (+)-nootkatone by bacteria was first found in 1970s (Dhavlikar & Albroscheit, 1973). (+)-Valencene was transformed to (+)-nootkatone by *Enterobacter* sp. with only 12% yield. Two Enterobacter strains isolated from Dutch soil and an infected German beer were proved to produce (+)-nootkatone. The maximum yield was 11 mol% (Dhavlikar & Albroscheit, 1973). There were also related studies on the transformation of other types of bacteria later. Okuda et al. used *Rhodococcus* species to produce (+)-nootkatone with a yield of 2.5 mg in 50-ml shake flasks. *Rhodococcus* KSM-5706 converted (+)-valencene into (+)-nootkatone with a yield of 0.5 mol% (50 mg/l), while it contained a lot of mixtures (Okuda, Sonohara, & Takikawa, 1994). Sowden et al. investigated the oxidation of (+)-valencene by wild type and mutants of P450_cam_ from *Pseudomonas putida* (Sowden et al., 2005). Wild type P450_cam_ could not oxidize (+)-valencene while the mutants had relevant activity to produce (+)-*trans*-nootkatol and (+)-nootkatone. It was also found that wild type P450_BM-3_ and mutants form *Bacillus megaterium* had higher activities than P450_cam_ but the selectivity was relatively low. It has been investigated as a potential route to produce (+)-nootkatone.

### Biotransformation of (+)-Valencene by Microalgae and Plant Cells

There were few studies on the biotransformation of terpenoids and aromatic flavor compounds by *Chlorella* species recently. Microalgae are important photosynthetic microorganisms and are also used for biotransformation of terpenes. Light energy is the only source of energy for microalgae growth. Therefore, the large-scale culture of microalgae is simpler and cheaper than other microorganisms. It was reported that sesquiterpene (+)-valencene was transformed to (+)-nootkatone by the green algae *Chlorella fusca* var. *vacuolata* IAMC-28 with a yield of 63% (252 mg/l) (Furusawa et al., 2005b). In contrast, *Chlorella prenoidosa* and *Chlorella vulgaris* had a better transformation effect. The yield of (+)-nootkatone exceeded 80% (320 mg/l) finally (Asakawa et al., 2013).

Some plant cells also have related transformation effects using (+)-valence as a precursor substance. Drawert and Berger investigated that the citrus tissue cultures had potential capacity to the biosynthesis of (+)-nootkatone with very small amounts (Drawert & Berger, 1983). Suspension cultures of *Citrus* sp. can convert (+)-valencene to (+)-nootkatone via the 2-hydroxy-derivative (nootkatol) (Drawert et al., 1984). It was found that (+)-nootkatone could be detected in three *Citrus* species, for example *Citrus paradisi, Citrus limonia*, and *Citrus aurantium* (Delrio et al., 1991; Reil & Berger, 1996). In addition, the study found that the suspension cultures of *Gynostemma pentaphyllum* could convert (+)-valencene to (+)-nootkatone through intermediate nootkatol. The incubation of (+)-valencene with *G. pentaphyllum* for 20 days obtained 72% (650 mg/l) yield of (+)-nootkatone. Furthermore, it was confirmed that the biotransformation could occur in two other cultured plant cells *Caragana chamlagu* and *Hibiscus cannabinus* (Sakamaki et al., 2005).

## Enzymes Associated with Microbial Transformation of (+)-Valencene to (+)-Nootkatone

The isolation, cloning, expression, and regulation of enzyme genes have become a hot research topic. It is expected to realize the industrial and efficient production of oxygen-containing terpenes aromatic compounds through genetic engineering technology. It was reported that some enzymes played an important role in the conversion process from (+)-valencene to (+)-nootkatone and its intermediate substance (+)-nootkatol, such as cytochrome P450 enzyme, laccase, oxidase, dehydrogenase, reductase, etc. (Table 2).

**Table 2.** Enzymes Associated with Microbial Transformation of (+)-Valencene to (+)-Nootkatone

Enzymes	Origin	Reference
**CYP_450_**		
P450_cam_	*Pseudomonas putida*	Sowden et al. (2005)
P450_BM-3_	*Bacillus megaterium*	Sowden et al. (2005)
CYP109B1	*Bacillus subtilis*	Girhard et al. (2009)
CYP71D55 (HPO)	*Hyoscyamus muticus* premnaspirodiene	Gavira et al. (2013), Takahashi et al. (2007)
CYP71AV8	Chicory	Cankar et al. (2011)
CYP71D51v2	Tobacco (*Nicotiana tabacum*)	Gavira et al. (2013)
CnVO (CYP706M1)	Alaska cedar (*Callitropsis nootkatensis*)	Cankar et al. (2014)
**Laccase**		
Laccase	*Botrytis cinerea*	Huang et al. (2001)
Laccase	*Funalia trogii*	Kolwek et al. (2018)
**Oxidase**		
Lignin peroxidase	*Phanerochaete chrysosporium*	Willershausen (1996)
Lipoxygenase	*Pleurotus sapidus*	Fraatz et al. (2009), Krugener et al. (2010)
Lipoxygenase	*Pleurotus florida*	Omarini et al. (2014)
**Dehydrogenase**		
Alcohol dehydrogenase (ADH-C3)	*Pichia pastoris*	Wriessnegger et al. (2014)
Glucose dehydrogenase (GDH)	*Bacillus megaterium*	Schulz et al. (2015)
Alcohol dehydrogenase (ADH1)	*Saccharomyces cerevisiae*	Ouyang et al. (2019)
Cytochrome P450 reductase (ATR1)	*Arabidopsis thaliana*	Meng et al. (2020)
Short-chain dehydrogenase/reductase (SDR) superfamily dehydrogenases ZSD1	*Zingiber zerumbet*	Meng et al. (2020)
Short-chain dehydrogenase/reductase (SDR) superfamily dehydrogenases A2B2	*Citrus sinensis*	Meng et al. (2020)
Cytochrome P450 reductase (CPR)	*Arabidopsis thaliana*	Cankar et al. (2011), Guo et al. (2018), Ouyang et al. (2019), Wriessnegger et al. (2014)
BMD_2094	*Bacillus megaterium*	Milhim et al. (2019)
**Reductase**		
Putidaredoxin reductase (PdR)	*Pseudomonas putida*	Girhard et al. (2009)

### CYP450s

Cytochrome P450s (CYPs) are a common enzyme in the microbial transformation. They are a kind of monooxygenated superfamily containing iron heme, which are widely distributed in bacteria, fungi and higher organisms. Cytochrome P450s (CYPs) are usually involved in a series of post-modifications of the core structure of terpenoids in a large part, resulting in the final terpenoid products with a wide range of chemical diversity (Mele et al., 2020; Xiao et al., 2019). Engineered cytochrome P450_cam_ and P450_BM-3_ were shown to transform (+)-valencene to (+)-nootkatone (Sowden et al., 2005). The CYP109B1 from *Bacillus subtilis* can catalyze the oxidation of (+)-valencene to produce nootkatol and (+)-nootkatone (Girhard et al., 2009). Plant cytochromes P450 were considered to be involved in the biosynthesis of (+)-nootkatone by metabolizing (+)-valencene. The premnaspirodiene oxygenase from *Hyoscyamus muticus* (HPO) CYP71D55 possess the ability to oxidize (+)-valencene to β-nootkatol *in vitro* (Gavira et al., 2013; Takahashi et al., 2007). Cankar et al. screened and cloned a cytochrome P450 mono-oxygenase (CYP71AV8) from chicory, which was capable of catalyzing the regioselective oxidation of (+)-valencene. This can also be used for the biotechnological production of (+)-nootkatone (Cankar et al., 2011). In addition, CYP71D51v2 from tobacco (*Nicotiana tabacum*) was reported to oxidize (+)-valencene predominantly to β-nootkatol. CYP71D51v2 and a P450 reductase from *Arabidopsis* expressed in the recombinant yeast brought about the production of β-nootkatol and (+)-nootkatone (Gavira et al., 2013). Moreover, studies have found that the formation of (+)-nootkatone from β-nootkatol was not rely on CYP450 and it was catalyzed by the yeast components. Besides, (+)-nootkatone was produced by coexpressing of a *Callitropsis nootkatensis* valencene oxidase (CnVO) CYP706M1 and a valencene synthase (CnVs) in yeast with a yield of 144 ± 10 μg/l (Cankar et al., 2014).

### Laccase

Laccases are oxidases mainly produced by basidiomycete fungi (Calcaterra et al., 2008). It is a copper-containing polyphenol oxidase that can oxidize polyphenols, methoxy-substituted phenols, diamines, and a large number of other compounds, but it cannot oxidize tyrosine (as do the tyrosinases) (Minussi et al., 2002). (+)-Valencene and a composition having laccase activity were reacted in the presence of an oxygen source to form (+)-valencene hydroperoxide, which was degraded to generate (+)-nootkatone (Huang et al., 2001). Laccases have been found to have a lot of application functions, such as bleaching in the textile and dye industry, production of wood composite materials, and the role of bioremediation (Linke et al., 2005). The method of producing (+)-nootkatone by laccase catalyzed oxidation of (+)-valencene was shown in Fig. 1b. The catalyzed process from (+)-valencene to (+)-nootkatone was two-steps and the intermediate product was valencene 2-hydroperoxides. (+)-Nootkatone was then produced by heating or adding a chemical catalyst. Laccase isolated from the basidiomycete *Funalia trogii* and dye-decolorizing peroxidase (Ftr-DyP) were confirmed to converted (+)-valencene to (+)-nootkatone with the highest concentration of 1,100 mg/l (Kolwek et al., 2018).

### Oxidase

Willershausen et al. proposed a cell-free enzymatic generation of (+)-nootkatone with isolated lignin peroxidase, and (+)-valencene was considered as the substrate under alkaline and high temperatures conditions (Willershausen, 1996). Krings et al. thought that some common terpene substrates, like (+)-valencene might likewise be catalyzed by dioxygenases (Krings et al., 2009). Afterwards, Fraatz et al. purified the responsible oxygenase from lyophilisates of *P. sapidus*, which could selective and highly efficient oxidation of (+)-valencene to (+)-nootkatone. The oxygenase had 50% homologies with the putative lipoxygenases from *Aspergillus fumigatus* and *Laccaria bicolor*. According to database research, it also had 26% homology with the sequence of lipoxygenase-1 from soy bean (Fraatz et al., 2009). Later, it was found that (+)-valencene was oxidized to hydroperoxides and the homologous data of key enzymes was confirmed, indicating a kind of lipoxygenase enzyme (Fig. 1c) (Krugener et al., 2010). The oxidation was found to proceed in the highly special region, especially at C7. One of the two equal allylic positions of the ring double bond of the (+)-valencene was oxidized. There was a correlation between the activity of lipoxygenase and the production of (+)-nootkatone. The hydroperoxides [2,2′,3,3′] of (+)-valencene were the intermediate products of the oxidation process and the final products were β-nootkatol, α-nootkatol, and nootkatone. The gene of lipoxygenase was also cloned and expressed heterogeneously in *Escherichia coli* to produce (+)-nootkatone (Zelena et al., 2012). Moreover, classical genetics approach had changed and partially improved the 60% terpene transformation capability and lipoxygenase activity of *Pleurotus* species (Omarini et al., 2014). The dioxygenases could create catalysts in the future engineering. Some terpenes and alkenes can be catalyzed, making them the precursors of new flavor compounds.

### Dehydrogenase and Reductase

In addition, there were some dehydrogenase and reductase enzymes involved in the conversion of (**+**)-valencene to (**+**)-nootkatone. Cytochrome P450 monooxygenase P450 _BM3_ could catalyze (**+**)-valencene to the intermediate alcohol nootkatol, which was further oxidized to (**+**)-nootkatone by an alcohol dehydrogenase (ADH). Regeneration of cofactors supported by glucose dehydrogenase (GDH) can be achieved regardless of whether NADPH or NADH was used (Schulz et al., 2015). Cytochrome P450 oxidoreductases (CPR) acted as electron donors and it worked in coordination with P450s monooxygenase (Bak et al., 2011). Milhim et al. identified a novel short chain dehydrogenase (SDR) from *Bacillus megaterium*, which could convert (*trans*)-nootkatol to (+)-nootkatone for the first time (Milhim et al., 2019). Putidaredoxin reductase (PdR) was used as a coexpression enzyme in heterologous expression (Girhard et al., 2009).

## Synthesis of (+)-Nootkatone by Heterologous Synthesis

**Fig. 2.**
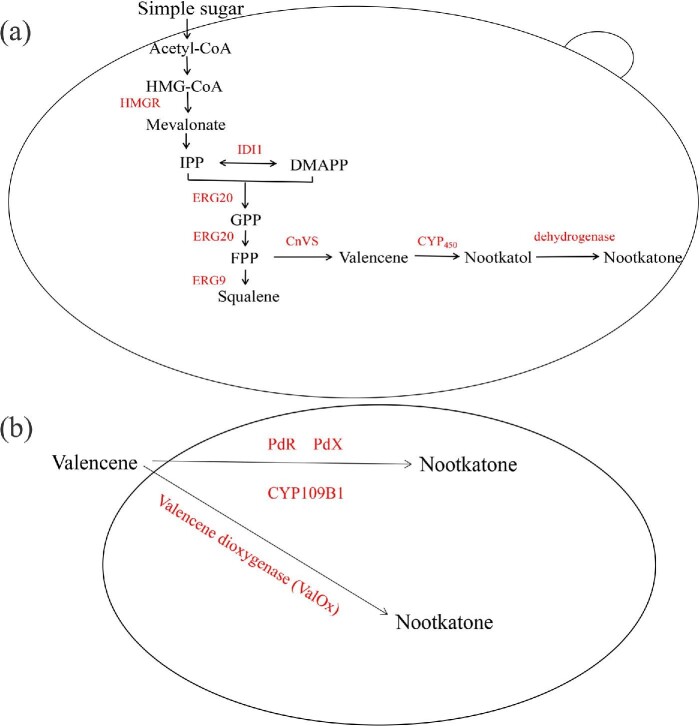
Heterologous synthesis of (+)-nootkatone in yeast (a) and *E. coli* (b). HMG-CoA, 3-hydroxy-3-methylglutaryl coenzyme A; IPP, isopentenyl pyrophosphate; DMAPP, dimethylallyl pyrophosphate; GPP, geranyl diphosphate; FPP, farnesyl diphosphate; HMGR, HMG-CoA reductase; IDI1, isopentenyl pyrophosphate (IPP) isomerase; ERG20, farnesyl diphosphate synthase; ERG9, squalene synthase enzyme; CnVS, (+)-valencene synthase.

In recent years, the metabolic engineering of microorganisms has made significant progress. Constructing microbial cell factories and heterologous expression of related enzymes became a great promising method for the production of (+)-nootkatone in yeast and *E. coli* (Fig. 2) (Table 3) (Paramasivan & Mutturi, 2017). The common precursor for terpenoid synthesis is isopentenyl diphosphate (IPP), which comes from Mevalonate pathway (MVA) or methyl-d-erythritol phosphate pathway (Maury et al., 2005). Condensation of IPP and its isomers dimethylallyl pyrophosphate (DMAPP) produce farnesyl diphosphate (FPP), which is the precursors for sesquiterpene synthesis. Currently, the plant (+)-valencene synthase was expressed in a microbial host and combined with the endogenous FPP delivery pathway and (+)-valencene oxidase. This method obtained (+)-nootkatone with “natural” properties in accordance with current food legislation.

**Table 3.** Heterologous Synthesis of (+)-Nootkatone

Heterologous expression vector	Enzymes	Nootkatone (maximum yield)	References
*Escherichia coli*	Valencene synthase gene from *C. sinensis*	/	Chappell and Greenhagen (2010), Sharon-Asa et al. (2003)
*Escherichia coli*	PdR; putidaredoxin (Pdx); CYP109B1	120 mg/l	Girhard et al. (2009)
*Escherichia coli*	Valencene dioxygenase (ValOx) from *Pleurotus sapidus*	80 mg/l	Zelena et al. (2012)
*Pichia pastoris*	(+)-Valencene synthase (CnVS); HPO; CPR; alcohol dehydrogenase (ADH)	208 mg/l	Wriessnegger et al. (2014)
*Yarrowia lipolytica ATCC 201249*	CnVS; CYP706M1; codon-optimized NADPH-cytochrome P450 reductase opAtCPR1	978.2 μg/l	Guo et al. (2018)
*Yeast WAT11*	CnVS; CYP71AV8; CPR	0.04 mg/l	Cankar et al. (2011)
*Saccharomyces cerevisiae*	CYP71D51v2 from tobacco; P450 reductase from *Arabidopsis*	4 mg/l	Gavira et al. (2013)
*Yeast WAT11*	CnVS; CYP706M1	144 ± 10 μg/l	Cankar et al. (2014)
*Saccharomyces cerevisiae W303*	(+)-Valencene synthase ValS; tHMG1; HPO; CPR; ICE2	/31 mg/l (total terpene)	Emmerstorfer et al. (2015)
*Saccharomyces cerevisiae*	CnVS; HPO; cytochrome P450 reductase from *Arabidopsis thaliana* (AtCPR); alcohol dehydrogenase (ADH1)	53.7 mg/l	Ouyang et al. (2019)
*Saccharomyces cerevisiae*	CnVS; HPO; ATR1; dehydrogenase/reductase (SDR) ZSD1	59.78 mg/l	Meng et al. (2020)

“/” represent there is no reports in literature.

In previous study, the researchers isolated a valencene synthase gene from *C. sinensis*, which was effective functionally expressed in *E. coli* and it could also be used for the subsequent production of (+)-nootkatone (Chappell & Greenhagen, 2010; Sharon-Asa et al., 2003). Afterwards, the regioselective oxidation test of (+)-valencene to (+)-nootkatone was carried out on 125 kinds of cytochrome p450 enzymes in bacteria. Studies have found that CYP109B1 of *B. subtilis* could catalyze the oxidation of (+)-valencene in *E. coli* with coexpression of PdR and putidaredoxin (Pdx) from *P. putida* (Girhard et al., 2009). Valencene dioxygenase (ValOx) from *P. sapidus* was expressed in the cytosol and periplasm of *E. coli*, which converted (+)-valencene to (+)-nootkatone and nootkatol through intermediate hydroperoxides (Zelena et al., 2012). Various strategies used by cold shock expression, partner coexpression and mutant *E. coli* strains improved the yield of soluble recombinant protein.

More recently, *Pichia pastoris* have become one of the biochemical hosts with a wide range of microorganisms (Siripong et al., 2020). Wriessnegger et al. coexpressed the HPO and CPR in *P. pastoris* with the addition of (+)-valencene (Wriessnegger et al., 2014). (+)-Valencene could also be produced in the cell through the coexpression of (+)-valencene synthase, which solved the phase transfer problem of (+)-valencene. Then, it was found that the yield of (+)-nootkatone reached 208 mg/l by additional overexpression of *P. pastoris* ADH and truncated hydroxy-methylglutaryl-CoA reductase. In addition, they also found that the overexpression of gene RAD52 had a significant positive effect on the formation of trans-nootkatol, which increased the production of *trans*-nootkatol (β-nootkatol) by five times compared with the original strain and conditions (Wriessnegger et al., 2016). *Y. lipolytica* was regarded as nonpathogenic and used as a production host for a large variety of biotechnological applications in several industrial processes classified as safe (Groenewald et al., 2014). The study achieved the heterologous production of (+)-nootkatone in *Y. lipolytica* by coexpressing CnVS, CYP706M1 and CPR. The final engineered strain produced (+)-nootkatone with an amount of 978.2 μg/l. This strain achieved the fusion of opCYP706M1 and opt46AtCPR1, and overexpressed the rate limiting enzymes tHMG1 and FPP synthase ERG20 in MVA pathway (Guo et al., 2018). The *S. cerevisiae* is a favorable host for the production of terpene compounds. It possessed the robustness of the strain, the compatibility with the existing infrastructure, and the performance of the availability of the existing genetic engineering molecular tools (Ignea et al., 2014). (+)-Nootkatone was produced by overexpressing (+)-valencene synthase and CYP71AV8 in yeast strain *WAT11*. The volume yields of (+)-valencene and (+)-nootkatone were 1.36 and 0.04 mg/l, respectively (Cankar et al., 2011). In addition, CYP71D51v2 and P450-reductase were cloned and expressed in *S. cerevisiae* (Gavira et al., 2013). Another study described the coexpression of CYP706M1 and (+)-valencene synthase in yeast strain *WAT11* to produce (+)-nootkatone. According to previous reports, 144 ± 10 μg/l (+)-nootkatone was produced and it had fewer intermediate products or by-products (Cankar et al., 2014). The total terpene yield reached 31 mg/l by heterologous expression of several key enzymes in the synthesis pathway of (+)-nootkatone in *S. cerevisiae w303* (Emmerstorfer et al., 2015). Related research has used *S. cerevisiae* as the host strain to reconstruct its metabolic pathway *in vivo* and biosynthesize (+)-valencene and its derivatives (+)-nootkatone. Finally, the synthesis of (+)-nootkatone reached 53.7 mg/l through resting cell transformation (Ouyang et al., 2019). Recently, a biosynthetic pathway was constructed in *S. cerevisiae* to produce the (+)-nootkatone by overexpressing the CnVS, HPO, and ZSD1 combined with the MVA pathway engineering. The maximum yield of (+)-nootkatone reached 59.78 mg/l. At the same time, it provided a solid foundation for the whole-cell production of (+)-nootkatone (Meng et al., 2020).

## Conclusions and Future Perspectives

The aroma and fragrance of food will increase our sense of pleasure, arouse appetite, promote saliva secretion, and enhance the digestion and absorption of nutrients. Therefore, flavors and fragrances are an indispensable part of food. Terpenoids are the most used spices at present, and oxygen-containing sesquiterpene (+)-nootkatone not only has unique aromatic odor, but also has important pharmacological effects such as antioxidant, antibacterial, and antitumor. In terms of academic significance and application value, biotransformation has unlimited prospects in the modification and transformation of sesquiterpene (+)-nootkatone. At present, researchers have conducted more research on biological transformation synthesis of natural spices. More attention should be paid on the directional transformation mechanism and regulation of (+)-valencene. The separation, cloning, expression and regulation of key enzyme genes in (+)-valencene transformation and (+)-nootkatone biosynthesis have become a hot topic. It is the focus of current research for discovering new and more efficient enzymes and improving systems for expressing them heterogeneously. Gene engineering technology is used to site-directed mutation of key enzymes, change the structure of enzyme molecules, so as to improve the activity of enzyme. Gene recombination technology can also be used to obtain genetically engineered strains with high expression of key enzymes. In addition, the selection of abundant and inexpensive substrates for microbial transformation to produce spices will have more application prospects. Therefore, reducing the production costs of natural flavor (+)-nootkatone and meeting the needs of consumers and producers through bioengineering technology are a practical technology.

## Data Availability

Experimental data are provided in the manuscript. Authors agree to provide any other data if requested.
